# Report of the Surgical Clinic of the Atlanta Medical College

**Published:** 1855-10

**Authors:** W. F. Westmoreland

**Affiliations:** Professor of Surgery


ARTICLE VII.—*Report of the Surgical Clinic of the Atlanta Medical College.* By W. F. WESTMORELAND, M. D., Professor of Surgery.

In the last number of this Journal we promised in this issue a continuation of the report of some of the more interesting cáses presented at the Surgical Clinic during the past session of the Atlanta Medical College.

CASE 1.—*Cataract—Operation by Absorption.*—Sampson, a man between 50 and 60 years of age, belonging to Mr. B. Tidwell, of Meriwether County, was sent to my Infirmary early in May for an operation. Several years ago, from some cause not now recollected, Sampson lost entirely his left eye, it being at the time of the examination completely atrophied.

Eight or ten months previous to my seeing him, his right eye commenced failing, which he at first attributed to age, but which steadily progressed, so that, for two months previous to his presenting himself to me he had not been able to see his way. Upon an examination, after dilating the pupil with a solution of Atropine, I found the lens almost perfectly opaque, there being only one small point, situated upwards and outwards, through which light could possibly reach the retina. From the color and other considerations I supposed the lens sufficiently hard to depress, and decided to operate by depression.

The day previous to the operation a dose of the Sulphate of Magnesia was administered, and immediately preceding it a quarter of a grain of the Sulphate of Morphine.

The pupil being well dilated, the needle (Scarpa's) was now introduced into the sclerotic coat, at the usual point, and after passing it in front of the lens, and lacerating the capsule, I attempted to depress it, but found the lens too soft—the needle passing through it at every attempt. I now decided to break up the lens and leave it for absorption, which I did by passing the needle several times through it.

I again introduced between the lids a few drops of a solution of Atropine, put the patient to bed, and enjoined perfect rest. Cold water, by means of cloths, was applied to the eye for the first three or four hours, after which ice, by means of oil-silk bags, was constantly applied for two or three days. No accident occurred, and at the expiration of six or eight days he could distinguish objects; in fifteen or twenty days he could see his way, and was improving so fast that I wrote Mr. T. that he might expect him at home in a week or ten days.

A few days after this, and between three and four weeks after the operation, he told me that he did not think he could see so well as he had the day previous. I paid but little attention to him at the time, thinking that perhaps the pupil was not so well dilated as it had been; but the next day he made the same remark. I now made a careful examination and found that the lacerated capsule was becoming opaque, and that at the points where the needle had passed through it plastic lymph had been thrown out and a false membrane formed, thus making a complete membrane stretched across the pupil, adhering at several points to the iris. It is necessary to add that the pupil, during the whole treatment, had been kept constantly dilated by means of a solution of Atropine.

The opacity continued to increase, and at the expiration of two or three weeks more the patient could not distinguish a man across the room. I determined to operate the second time, and the operation proposed was to cut loose, if possible, the capsule and membrane from its superior and lateral attachments to the iris, and depress it—placing it immediately behind the inferior portion of the iris. This I did in a similar case, the past winter, with complete success. But if, after introducing the needle, it was found impossible to detach the capsule, as above proposed, I determined to make a transverse incision through it, with the hope that it would become sufficiently large to admit the necessary amount of light upon the retina. I should have attempted its extraction, but for the apparent firm adhesions to the iris.

After deciding upon the operation, I introduced the needle, as in the operation by depression, and succeeded in passing it in front of the capsule—broke up readily its lateral attachments—but after several efforts failed in detaching the capsule from the superior portion of the iris. I now turned the cutting edge of the needle towards the capsule, and succeeded in making a complete section of it—leaving the superior and inferior portions floating behind the pupil. The section of the capsule was made rather nearer its superior than its inferior attachment, with the hope that by position the free margin of the inferior segment would descend, and thus admit the necessary amount of light into the posterior chamber.

The patient was now placed in a comfortable position, and cold water and ice applied, as in the first operation. All did well, and the day after the operation the patient could see very well. On the morning of the second day he could see as well as is usual after the removal of the lens. At night, on the second day after the operation, while the nurse was in an adjoining room, Sampson, from some cause unknown to me, got out of bed, dressed himself, and left for home, a distance of some fifty or sixty miles. He made between twenty and thirty miles of the distance on foot, and the remainder in some conveyance.

I have not seen the patient since he left the Infirmary, but learn that there resulted from the fatigue and exposure to a burning sun, a violent grade of inflammation of the eye, which, however, subsided at the expiration of twelve or fifteen days without destroying the eye. He still sees sufficiently to go about without a guide, but not enough to make himself useful upon a farm.

